# Expression of human epidermal growth factor receptor 2 in bladder urothelial carcinoma

**DOI:** 10.1186/s12907-017-0046-z

**Published:** 2017-04-04

**Authors:** Mohamed Reda El Ochi, Mohamed Oukabli, Elarbi Bouaiti, Hafsa Chahdi, Adil Boudhas, Mohamed Allaoui, Ahmed Ameur, Mohamed Abbar, Abderrahmane Al Bouzidi

**Affiliations:** 1 Department of Pathology, Mohamed V Military Hospital, Hay Riad, Rabat, Morocco; 2 Faculty of Medicine and Pharmacy, Mohammed V University, Hay Riad, Rabat, Morocco; 3 Laboratory of Biostatistics Clinical Research and Epidemiology, Mohamed V Military Hospital, Hay Riad, Rabat, Morocco; 4 Department of Urology, Mohamed V Military Hospital, Hay Riad, Rabat, Morocco; 5 Hôpital militaire Mohamed V, Hay Riad, BP10000 Rabat, Morocco

**Keywords:** Bladder, HER2, Carcinoma, Urothelial

## Abstract

**Background:**

Urothelial bladder carcinoma (UBC) is one of the most prevalent cancers in men worldwide. Human epidermal growth factor receptor 2 (HER2) expression has been detected in a wide range of urothelial carcinoma. Despite many reports in the literature, the prognostic significance of this overexpression remains unclear. The aim of this study was to assess the expression of HER2 in urothelial bladder carcinomas and its association with clinical and pathological parameters.

**Methods:**

103 cases of UBC were diagnosed in our department between January 2014 and December 2015. The tumor specimens obtained by transurethral resection or cystectomy were evaluated by immunohistochemistry using HER2 antibody.

**Results:**

HER2 protein overexpression was present in 11.7% of cases and associated with tumor grade (*p* = 0.003) and pathological stage (*p* = 0.015). In multivariate analysis, HER2 overexpression was associated only with tumor grade (*P* = 0.04).

**Conclusion:**

HER2 protein overexpression is noted in patients with high grade cancer. This expression may select patients for anti HER2 targeted therapy. Future larger and prospective studies will verify the frequency of HER2 alteration and the role of HER2 in the aggressive behavior.

**Electronic supplementary material:**

The online version of this article (doi:10.1186/s12907-017-0046-z) contains supplementary material, which is available to authorized users.

## Background

Urothelial carcinoma of the bladder (UCB) is the fourth most prevalent type of cancer in men worldwide [1] with 74,690 new cases and 15,580 deaths in 2014 in the USA [2]. It is a heterogeneous disease [3]. More than 75% of UCB is classified non muscle invasive bladder carcinoma [1]. Several factors involve in determination of prognosis and selection of treatment including patient’s age, grade, pathological stage and concomitant carcinoma in situ [4–6]. However, they may be insufficient in determination of the prognosis [7]. In fact, different outcomes are can be observed in patients at the same stage and grade [1]. In addition, therapeutic weapons are limited in metastatic UCB and permit only a small improvement [8]. Thereby, other factors such as molecular markers can be helpful for estimating the risk of progression and the response to alternative targeted therapies [7].

Human epidermal growth factor receptor 2 (HER2) is a transmembrane tyrosine kinase receptor who is involved in cell growth, survival and migration [9–11]. Increased activity of this molecule has been evaluated in breast cancer and was associated with a poor prognostic and response to target therapy with specific antibodies as trastuzumab [12, 13]. The encouraging results of antibody in breast and advanced gastric or gastro-oesophageal junction cancer have incited the study of HER2 expression in other cancers to provide the use of HER2 inhibitors [13]. A wide variability of HER2 overexpression in bladder cancer, from 6 to 80%, has been reported [14] generally related with high grade and stage and correlated with poor prognosis [15–17]. However, other studies have found no such association [14, 18, 19]. Therefore, the value of HER2 overexpression in UCB is still unclear.

The purpose of the current study was to evaluate the HER2 expression in UCB and its association with clinical and pathological factors.

## Methods

### Patients and tissue specimens

A cross-sectional study of UBC cases diagnosed in our department between January 2014 and December 2015 was conducted. The tumor specimens were obtained from 75 transurethral resections and 28 cystectomies. For cases that had endoscopic treatment, extensive resection of the detrusor was carried out and addressed separately to allow an accurate staging.

Four micrometer thick sections were stained with hematoxylin-eosin and reviewed again by one pathologist (MREO). Staging was performed using the seventh edition of the tumor-node-metastasis classification [20]. The 2004 world health organization classification was used for tumor grading [21]. Clinicopathological data including age, sex, tumor size, the presence of carcinoma in situ, papillary and micropapillary growth pattern were collected from medical and pathological reports (Additional file 1).

### Immunohistochemistry

**Fig. 1:**
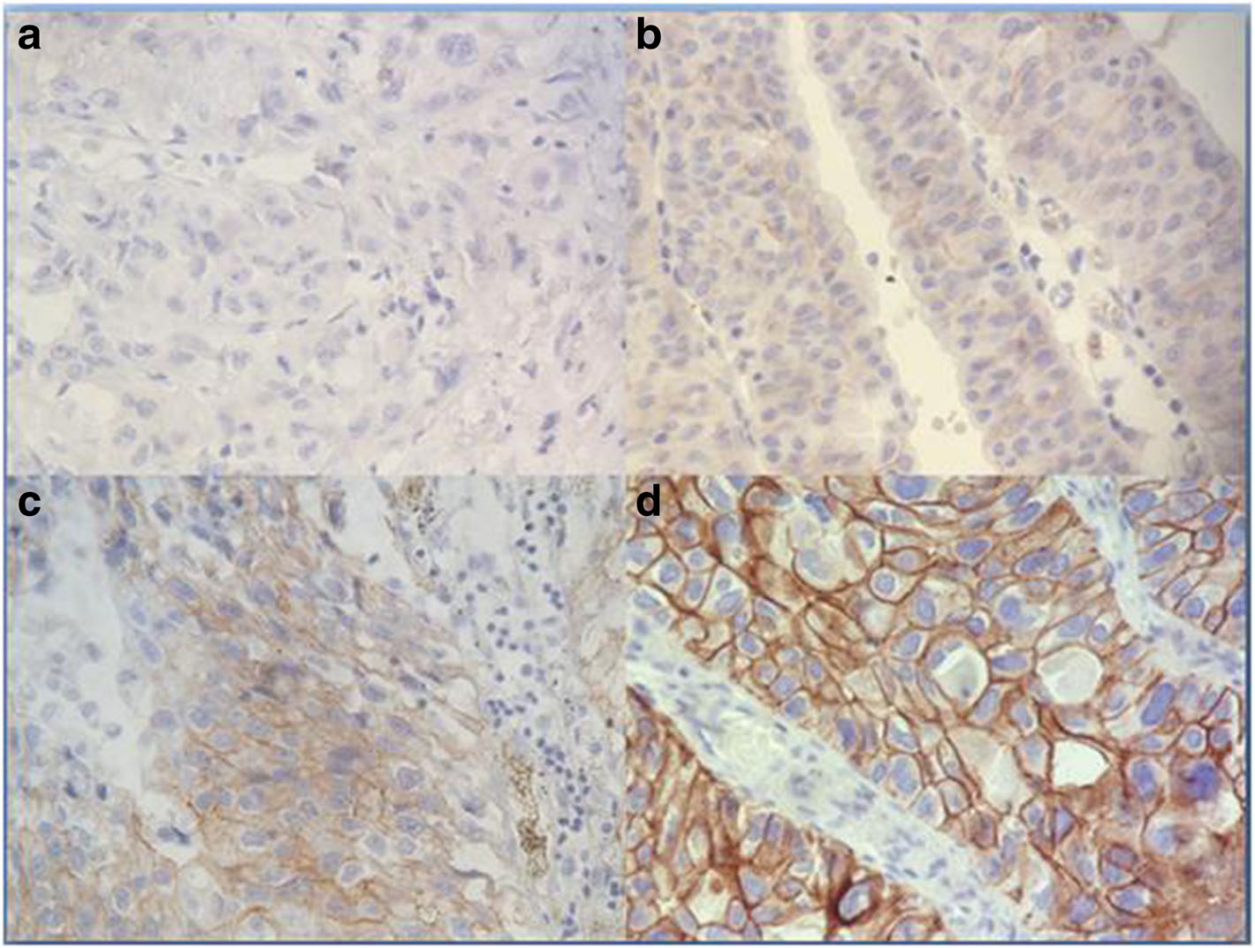
HER2 expression in urothelial bladder carcinoma: **a** HER2 negative staining scored as 0. **b** HER2 weak staining scored as 1+. **c** HER2 moderate staining scored as 2+. **d** HER2 positive staining scored as 3+

The hercepTest kit (K5204, Dako, Glostrup, Denmark) was used for HER2 protein expression analysis according to the manufacturer’s instructions. Briefly, deparaffinized tissue sections were first incubated in a 95 °C water bath for 40 min. Peroxydase-blocking solution was used for 5 min to prevent nonspecific immunostaining. Subsequently, the sections were incubated with the primary antibody, rabbit anti-human HER2 for 30 min and followed by the kit visualization reagent. The reaction product was visualized by the chromogen DAB. Positive and negative, external and internal controls were included for validation of the reactions. For Scoring, we used the recommendations of the American Society of clinical oncology/college of American Pathologists [22] (Fig. 1).

### Chromogenic in situ hybridization

Cases showing an immunostaining score of 2+ were tested by chromogenic in situ hybridization using HER2 pharmDX kit.

### Statistics

Statistical analysis was performed with the SPSS software (Statistical Package for the Social Sciences, version 13, SPSS Inc, Chigago, I 11, USA). The Mann-Whitney test, chi-square or Fisher exact test were used to evaluate the statistical significance of the associations between clinicopathological parameters. For multivariate analysis, binary logistic regression was performed incorporating significant parameters by the univariate analysis. Statistical significance was considered if *P* value was <0.05.

## Results

In the present study, 103 cases of UBC were included.

### Clinicopathological characteristics

The clinicopathological characteristics of the 103 patients are summarized in Table 1.

**Table 1:** Clinicopathological characteristics of the 103 patients with UBC

Characteristics	NO. (%) of cases
Age (median, interquartile range, years)	63 [57–74]
Sex	
Men	96 (93.2)
Women	7 (6.8)
Tumor size	
< 3 cm	43 (41.7)
≥ 3 cm	60 (58.3)
Papillary architecture	
Absent	36 (35)
Present	67 (65)
Carcinoma in situ	
Absent	101 (98.1)
Present	2 (1.9)
Tumor grade	
Low	68 (66)
High	35 (34)
Differentiation	
None	93 (90.3)
Micropapillary	3 (2.9)
Glandular	2 (1.9)
Squamous	5 (4.9)
T stage	
≤ pT1	82 (79.6)
> pT1	21 (20.4)
HER2 protein expression	
0	56 (54.4)
1+	32 (31.1)
2+	3 (2.9)
3+	12 (11.7)

The median age of the patients was 63 years. The patients included 96 men (93.2%) and the sex ratio was 13:1. Three centimeters or more sized carcinomas were found in 60 (58.3%). High grade was noted in 35 cases (34%). Eighty-two tumors (79.6%) had pathological stage ≤ T1 and 21 (20.4%) were > T1. Increased HER2 protein expression was present in 12 (11.7%) of 103 UBC. Three cases were scored as 2+ but without amplification in chromogenic in situ hybridization.

### Association of HER2 expression with clinicopathological parameters

We found statistically significant difference between HER2 overexpression and tumor grade (*p* = 0.003) and pathological stage (*p* = 0.015) (Table 2).

**Table 2:** Comparison of HER2 status with clinicopathological parameters of patients with UBC (*n* = 103)

	HER2 protein overexpression NO. (%) of cases		*P*-value
	Absent	Present	
Age (median, interquartile range, years)	64 [55.5–74]	60 [59.5–70.5]	0.85
Sex			
Men	85 (82.5)	11 (10.7)	
Women	6 (5.8)	1 (1)	0.59
Tumor size			
< 3 cm	40 (38.3)	3 (2.9)	
≥ 3 cm	51 (49.5)	9 (8.8)	0.21
Papillary architecture			
Absent	29 (28.2)	7 (6.8)	
Present	62 (60.2)	5 (4.8)	0.1
Carcinoma in situ			
Absent	89 (86.4)	12 (11.6)	
Present	2 (2%)	0 (0)	1
Tumor grade			
Low	65 (63.1)	3 (2.9)	
High	26 (25.2)	9 (8.7)	*0.003*
Micropapillary differentiation			
Absent	89 (86.3)	11 (10.7)	
Present	2 (2)	1 (1)	0.31
T stage			
≤ pT1	76 (73.8)	6 (5.8)	
>pT1	15 (14.6)	6 (5.8)	*0.015*

Italic: Statistically significant at *p* <0.05

In univariate analysis, HER2 overexpression was associated with grading (*P* = 0.004) and pathological staging (*P* = 0.012). In multivariate analysis considering tumor size, papillary and micropapillary pattern, carcinoma in situ, tumor grade and pathological stage, HER2 overexpression was correlated only with tumor grade (*P* = 0.042) (Table 3).

**Table 3:** Association of HER2 expression with clinicopathological parameters of patients with UBC (*n* = 103)

	Univariate analysis			Multivariate analysis		
	OR	IC 95%	*P*-Value	OR	IC95%	*P*-Value
Age	1.01	0.96–1.06	0.77			
Sex						
Women	1					
Men	1.29	0.14–11.7	0.82			
Tumor size						
< 3 cm	1			1		
≥ 3 cm	2.35	0.6–9.26	0.22	0.73	0.12–4.46	0.74
Papillary architecture						
Absent	1			1		
Present	0.33	0.1–1.14	0.081	0.93	0.18–4.9	0.94
Carcinoma in situ						
Absent	1			1		
Present	0.01	0.01-6.4E + 19	0.84	0.73	0.01-6.2E + 17	0.81
Tumor grade						
Low	1			1		
High	7.5	1.9–29.9	*0.004*	5.67	1.1–30.2	*0.042*
Micropapillary differentiation						
Absent	1			1		
Present	4.04	0.34–48.3	0.27	1.7	0.1–30.4	0.72
T stage						
≤ pT1	1			1		
>pT1	5.06	1.4–17.8	*0.012*	2.58	0.55–12.2	0.29

Italic: Statistically significant at *p* <0.05

## Discussion

UBC is the fourth most prevalent type of cancer in men worldwide with 74,690 new cases and 15,580 deaths in 2014 in the USA [1]. It’s a heterogeneous disease [3]. Outcomes can be different in patients at the same pathological stage or grade [1]. Prognostic factors are insufficient in determination of the prognosis [7]. Considerable attention has been given to the identification of prognostic biomarkers in UBC [1]. HER2 is a potential therapeutic target and one of the most frequently amplified oncogenes in bladder cancer [23]. It’s located on chromosome 17q21 and encodes a transmembrane protein that interacts with various growth factors. It is well known that the overexpression of HER2 protein is an important prognostic factor in breast carcinoma [15]. However, the role of HER2 in UBC remains unclear [15]. HER2 presents a potential prognostic factor and may led to HER2 targeted therapy to offer a survival advantage in patients with UBC [1]. A wide variability of HER2 protein overexpression (2 to 85%) has been reported in UBC and was associated with most advanced cancers and poor prognosis [1, 3, 13, 15], but the real frequency could be approximately 5–10% [18, 24]. This variation is due to the use of bladder cancer samples with a varying degree of histological grades and tumor pathological stages [3]. Indeed, HER2 status may differ among different ethnic or geographic populations [14]. Other reasons could be attributed to technical heterogeneity in immunohistochemistry, the use of different criteria for definition of positivity and antibody clones [18, 23, 25]. The evaluation of HER2 was generally carry out using the American Society of Clinical Oncology/College of American Pathologists guideline [11, 19] which has been updated in 2013 [13–15].

Most papers have shown that HER2 overexpression was significantly correlated with poor clinicopathological factors and poor outcome in UBC [3, 13, 16, 25]. Simonetti et al. and simon et al. reported association between HER2 statut and tumor grade and pathological stage [15, 23]. Others showed only association with tumor grade [1, 4, 26]. Zhao et al. found significant association between tumor grade and HER2 expression [1]. HER2 overexpression also correlated with lymph node metastases, recurrence and progression free survival [1, 3, 25, 26]. However, other studies have found no such association [14, 16].

The concordance between immunohistochemistry and in situ hybridization seem to be less strong than that found in breast cancer [14, 15, 23]. This discrepancy raises the question of what might be the optimal diagnostic procedure to select patients who may benefit from treatment [23]. Negative immunohistochemical staining demonstrated a high predictive value and specificity for negative gene amplification, suggesting that immunohistochemistry could be used as an initial screening tool to triage cases for in situ hybridization [3, 11, 14, 23].

## Conclusion

In summary, the role of HER2 status on UBC prognosis is still unclear. The overexpression of HER2 protein is noted in patients who appeared to have a more aggressive disease and therefore merits consideration. This expression should be determinate in high grade UBC and may select patients who are likely to benefit from anti HER2 targeted therapy. Future larger and prospective studies will be needed to ascertain the frequency of HER2 alteration and the role of HER2 in the aggressive behavior. An ongoing randomized phase III study on the maintenance lapatinib in UBC patients with HER2 2 to 3+ immunohistochemistry might shed additional lights.

## Additional file

Additional file 1: DATA EXCEL. data generated/analysed during the study. (XLSX 13 kb)

## Abbreviations

HER2: Human epidermal growth factor receptor 2
UBC: Urothelial bladder carcinoma
